# Supplementary material for: Metal oxide charge transfer complex for effective energy band tailoring in multilayer optoelectronics
Source: Nat Commun. 2022 Jan 10;13:75. doi: 10.1038/s41467-021-27652-3 (PMC8748812; doi:10.1038/s41467-021-27652-3)
Supplement: Supplementary file 1 — Supplementary Information [file 41467_2021_27652_MOESM1_ESM.pdf]

Supplementary Information

Metal Oxide Charge Transfer Complex for Effective Energy Band Tailoring in Multilayer Optoelectronics

Moohyun Kim[1], Byoung-Hwa Kwon[2], Chul Woong Joo[2], Myeong Seon Cho[1], Hanhwi Jang[1], Ye ji Kim[1], Hyunjin Cho[1], Duk Young Jeon[1], Eugene N. Cho[3,*], and Yeon Sik Jung[1,*]

[1] Department of Materials Science and Engineering, Korea Advanced Institute of Science and Technology (KAIST), 291 Daehak-ro, Yuseong-gu, Deajeon 34141, Republic of Korea

[2] Reality Device Research Division, Electronics and Telecommunications Research Institute (ETRI), 218, Gajeong-ro, Yuseong-gu, Daejeon 34129, Republic of Korea

[3] KAIST Institute for NanoCentury, Korea Advanced Institute of Science and Technology (KAIST), Daehak-ro, Yuseong-gu, Daejeon 34141, Republic of Korea

These authors contributed equally: Moohyun Kim, Byoung-Hwa Kwon

[*] Corresponding authors:

Email: nmecho@kaist.ac.kr (E. N. Cho), ysjung@kaist.ac.kr (Y. S. Jung)

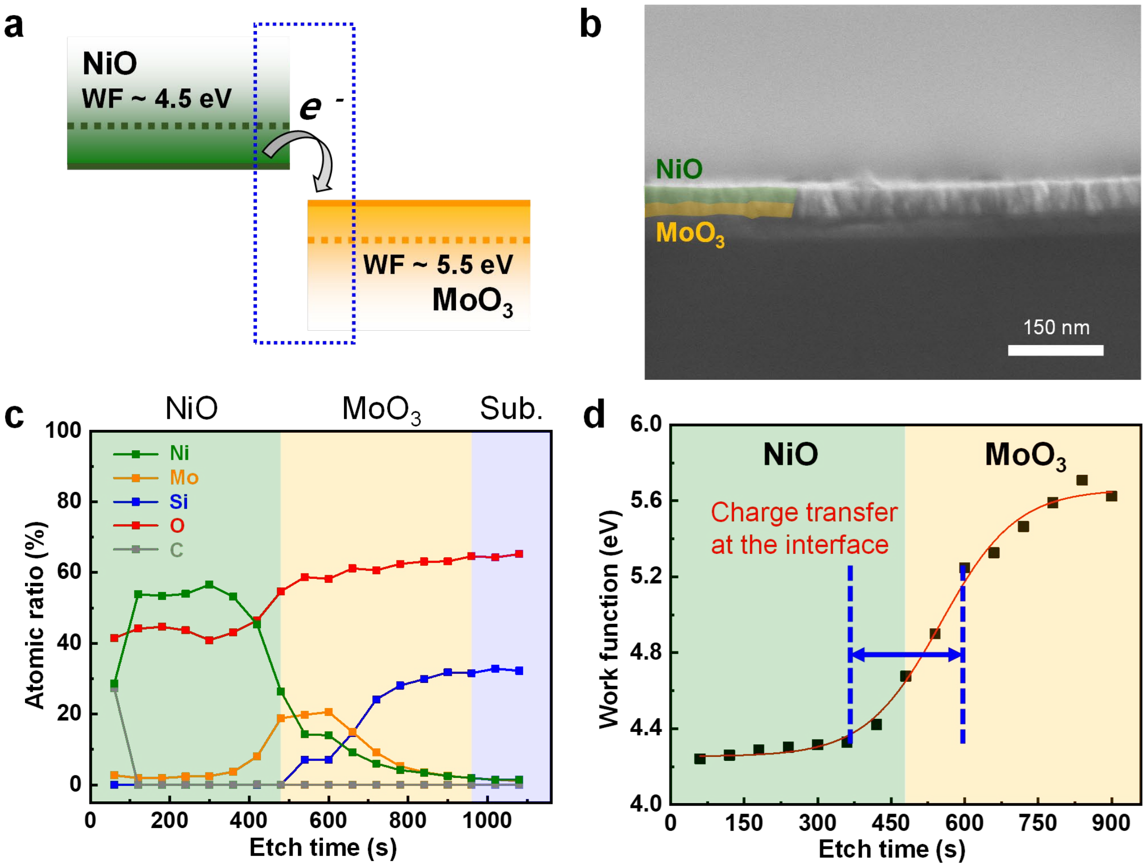

Supplementary Fig. 1. Energy level modulation at the interface of NiO/MoO_3 bilayer thin films (SCTD system). a Schematic of NiO/MoO_3 energy configuration and charge transfer phenomenon at the interface of two metal oxides. **b** SEM image of NiO/MoO_3 bilayer thin films. Each layer with a thickness of 20 nm was e-beam deposited. **c** XPS depth profiling of NiO/MoO_3 bilayer thin films. **d** Work function shifts of NiO/MoO_3 bilayer thin films depending on etching time.

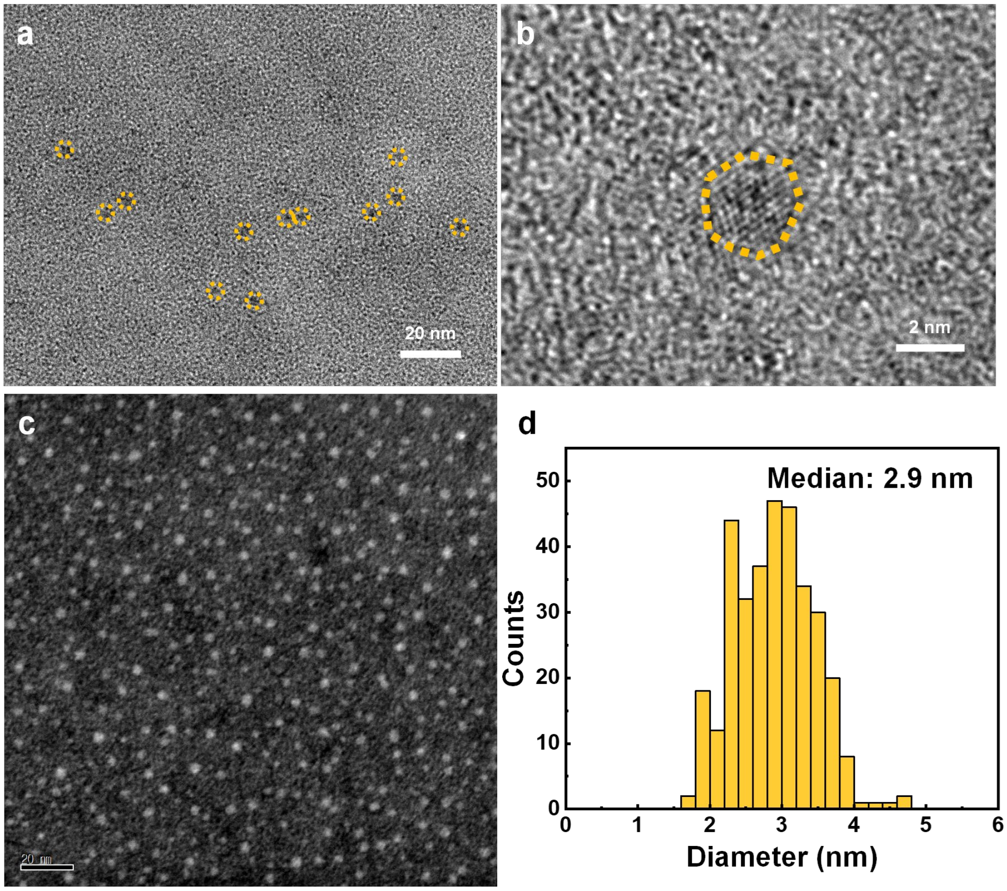

Supplementary Fig. 2. Size and distribution of monodisperse MoO_3 NPs. HRTEM images of **a** MoO_3 NPs (scale bar: 20 nm) and **b** a magnified image (scale bar: 2 nm) showing the lattice fringes of the NPs. HAADF-STEM image of **c** MoO_3 NPs (scale bar: 20 nm) and **d** their size distribution histogram.

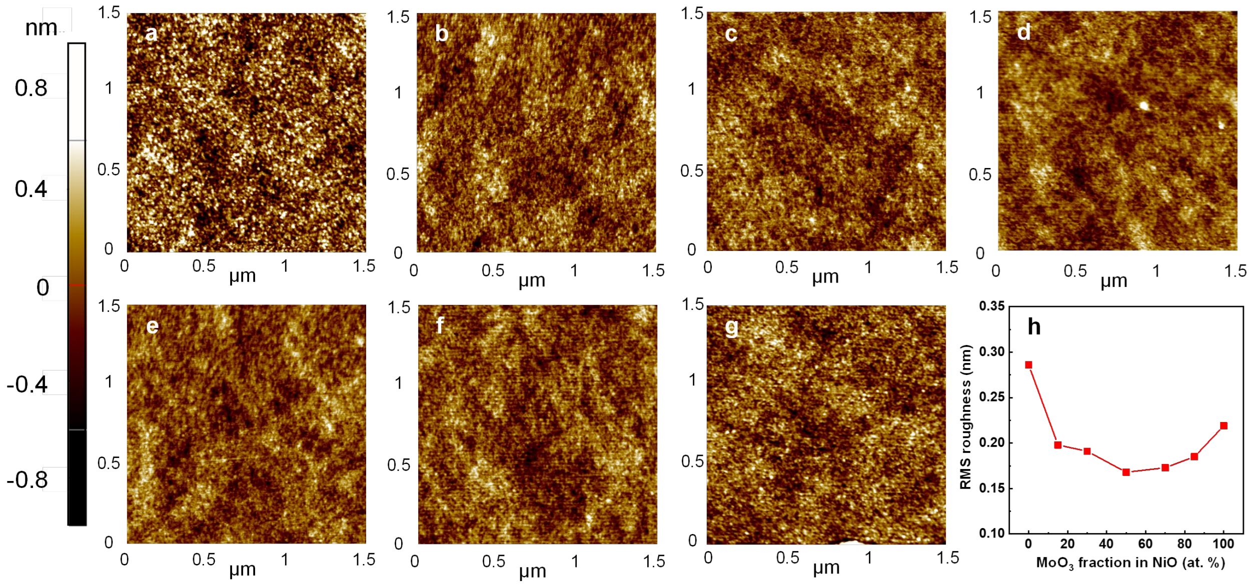

Supplementary Fig. 3. Surface roughness properties of each films. Atomic force microscopy (AFM) images (1.5 × 1.5 μm of scan area) of **a** NiO, **b** NiO:MoO_3 15 at.%, **c** NiO:MoO_3 30 at.%, **d** NiO:MoO_3 50 at.%, **e** NiO:MoO_3 70 at.%, **f** NiO:MoO_3 85 at.%, and **g** MoO_3. **h** Their root mean square (RMS) roughness values are 0.286 nm, 0.198 nm, 0.191 nm, 0.168 nm, 0.173 nm, 0.185 nm, and 0.219 nm for NiO, NiO:MoO_3 15 at.%, NiO:MoO_3 30 at.%, NiO:MoO_3 50 at.%, NiO:MoO_3 70at.%, NiO:MoO_3 85 at.%, and MoO_3, respectively.

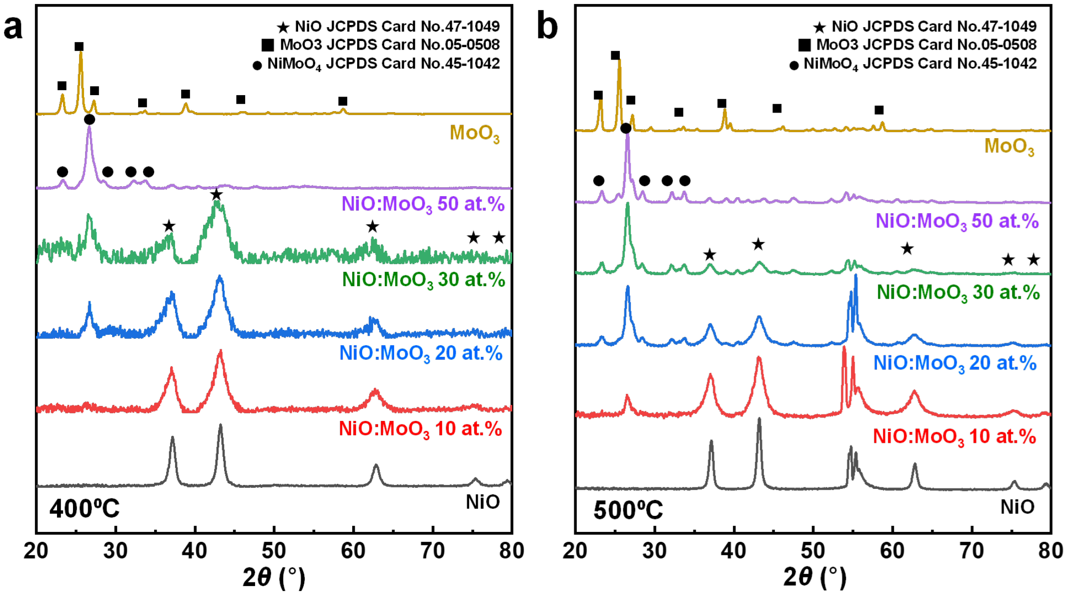

Supplementary Fig. 4. XRD patterns of NiO:MoO3-complex. a 400˚C and **b** 500˚C annealing temperatures for the NiO:MoO3-complex with varying MoO3 compositions.

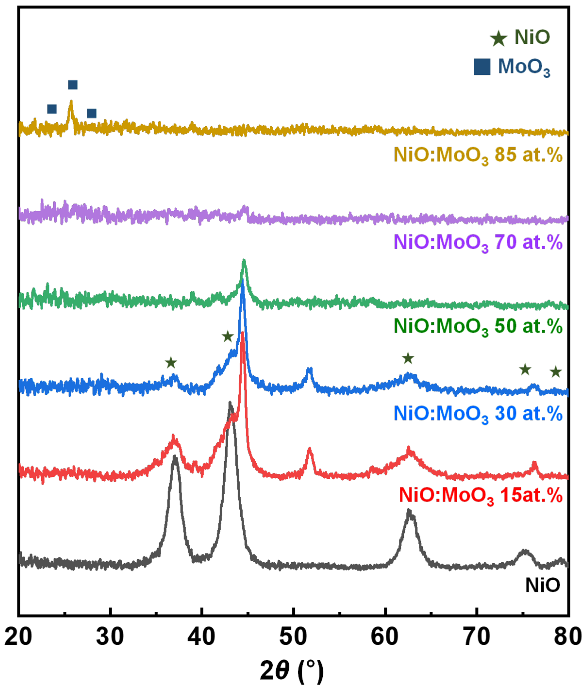

Supplementary Fig. 5. XRD patterns of the NiO:MoO_3-complex fabricated with various MoO_3 NP ratios at 300 °C.

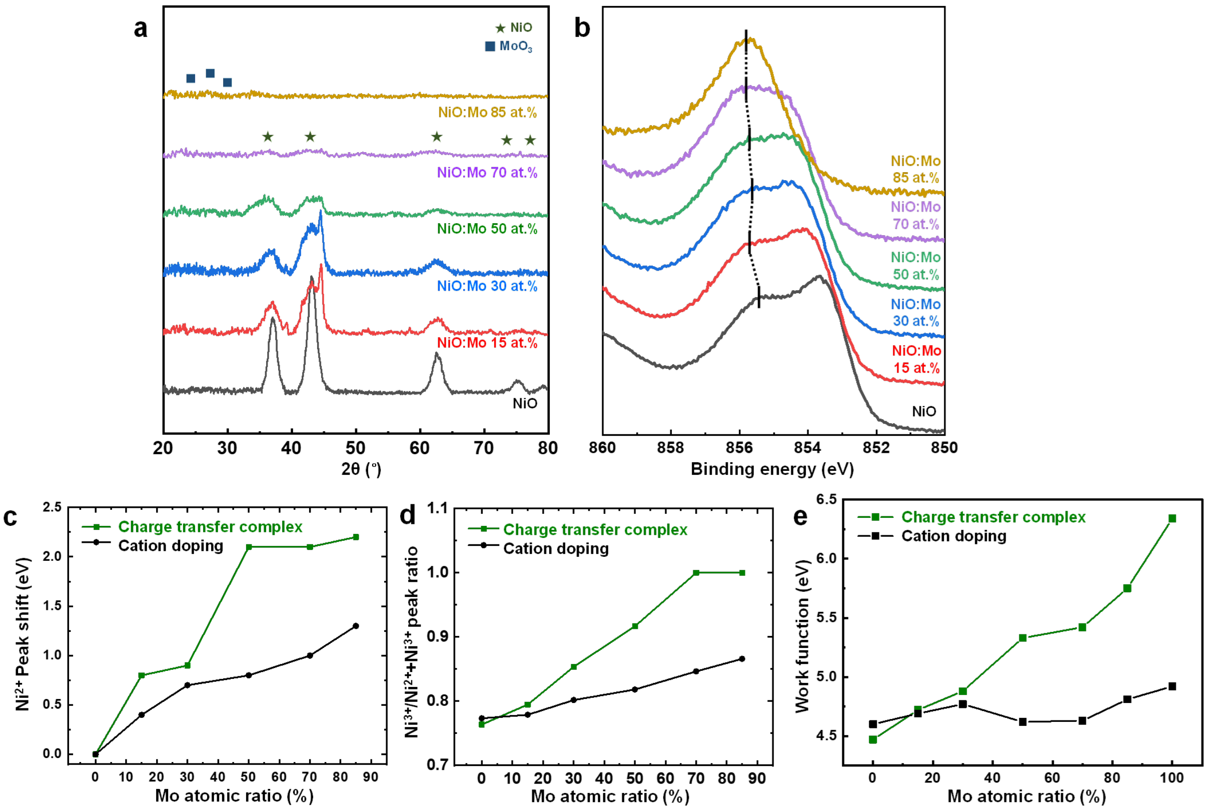

Supplementary Fig. 6. Characterization of Mo-doped NiO and comparison with the NiO:MoO3-complex. XRD patterns of **a** Mo-doped NiO with various Mo fractions. XPS studies of Mo-doped NiO. **b** Ni 2*p*5/2 narrow scan spectra of conventional Mo-doped NiO. Comparisons between the NiO:MoO3-complex and the Mo-doped NiO **c** Ni^{2+} peak shift and **d** $Ni^{3+}/Ni^{2+}+Ni^{3+}$ peak ratios depending on the Mo atomic ratio. **e** Measured work functions of NiO:MoO3-complex and Mo-doped NiO films.

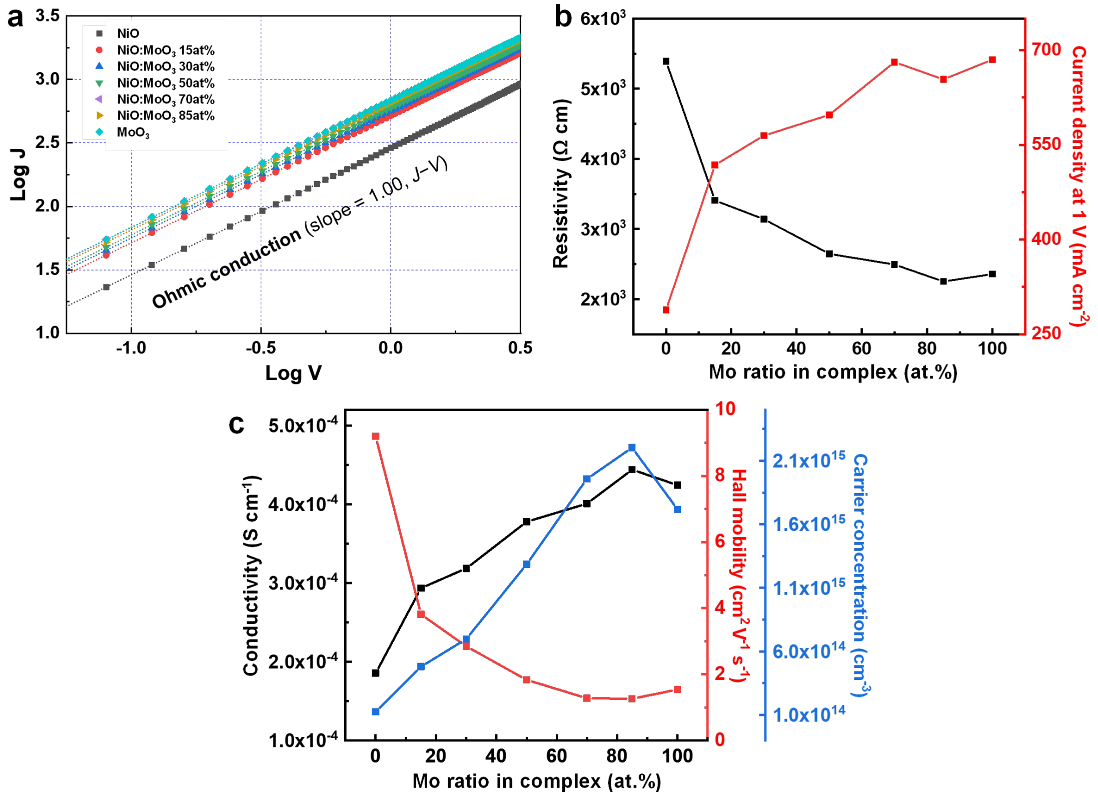

Supplementary Fig. 7. Electrical conductivity trends of NiO:MoO3-complex thin film. a Log J–log V plot of *J–V* characteristic (Fig. 3f) in ITO/thin film/Au device of NiO, MoO_3, and NiO:MoO_3-complex. Dashed lines are the linear fit of each device, following the ohmic conduction regime (slope = 1.00). **b** Comparison of resistivity conducted by the Hall measurement system and current density at 1 V from *J–V* curve (Fig. 3f) of NiO, MoO_3 and NiO:MoO_3 complex thin film. **c** Electrical conductivity, Hall mobility, and carrier concentration of NiO, MoO_3, and NiO:MoO_3 complex thin film obtained from Hall effect measurement.

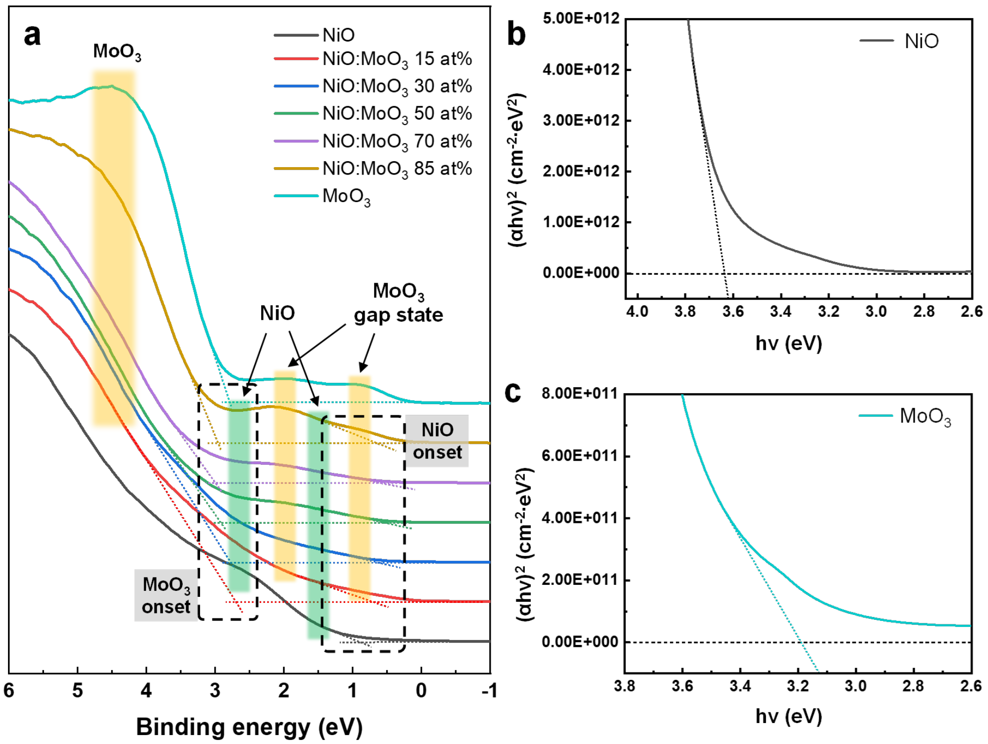

Supplementary Fig. 8. Ultraviolet photoelectron spectroscopy (UPS) spectra and Tauc plot for energy level evaluation. a Valence band onset spectrum of NiO, NiO:MoO_3-complex, and MoO_3. Tauc plot of **b** NiO and **c** MoO_3. Valence band onset points of NiO and MoO_3 were counted separately because the NiO:MoO_3-complex is not an alloy but a distinct metal oxides similar to an organic charge transfer complex.

Supplementary Table 1. Electronic energy levels and barrier heights with electrodes of NiO, MoO_3, and NiO:MoO_3-complex.

		NiO	NiO:MoO_3 15 at. %	NiO:MoO_3 30 at. %	NiO:MoO_3 50 at. %	NiO:MoO_3 70 at. %	NiO:MoO_3 85 at. %	MoO_3
Work-function (eV)		4.47	4.72	4.88	5.33	5.42	5.75	6.34
NiO	Valence band shift (eV)	0.9	0.7	0.64	0.56	0.6	0.64	–
	Valence band edge (eV)	5.37	5.42	5.52	5.89	6.02	6.39	
	Bandgap (optical, eV)	3.64						
	Conduction band edge (eV)	1.73	1.78	1.88	2.25	2.38	2.75	
MoO_3	Valence band shift (eV)	–	2.7	2.77	2.92	3	2.96	2.8
	Valence band edge (eV)		7.42	7.65	8.25	8.42	8.71	9.14
	Bandgap (optical, eV)		3.19					
	Conduction band edge (eV)		4.23	4.46	5.06	5.23	5.52	5.95
NiO E_v – ITO (eV)		0.67	0.72	0.82	1.19	1.32	1.69	–
ITO – MoO_3 E_c (eV)		–	0.47	0.24	-0.36	-0.53	-0.82	-1.25
NiO E_v – Au (eV)		0.27	0.32	0.42	0.79	0.92	1.29	–
Au – MoO_3 E_c (eV)		–	0.87	0.64	0.04	-0.13	-0.42	-0.85

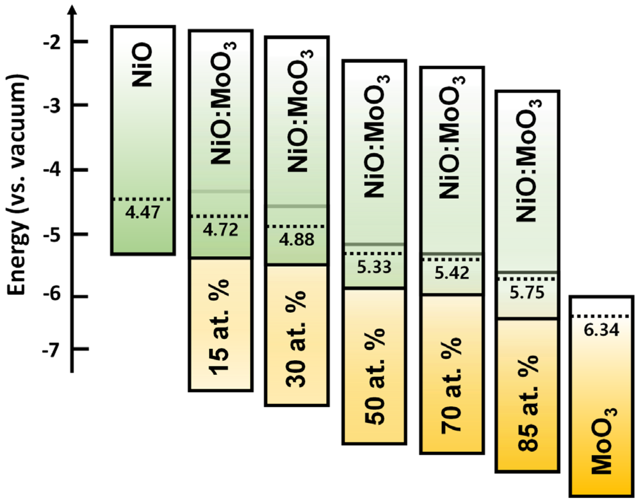

Supplementary Fig. 9. Energy diagrams of the NiO, MoO_3, and NiO:MoO_3-complexes with varied MoO_3 composition.

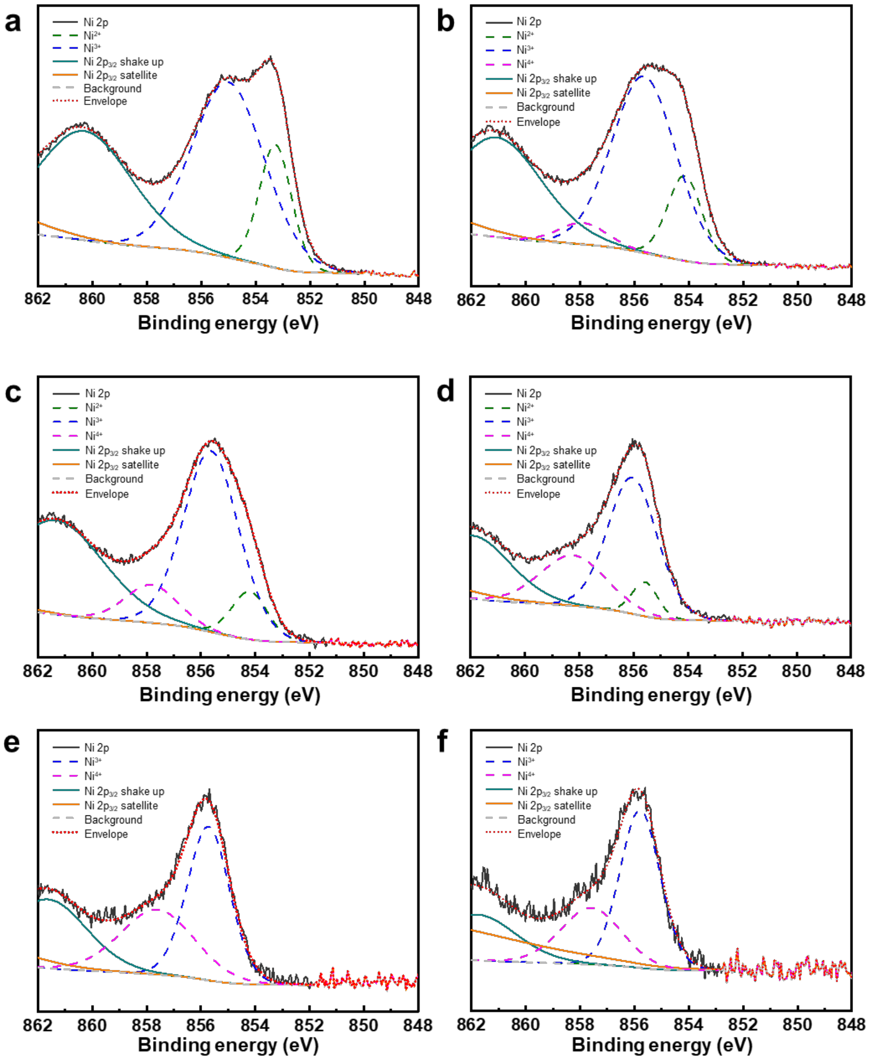

Supplementary Fig. 10. Deconvolution of XPS Ni 2*p* narrow scan spectra of pristine NiO and NiO:MoO_3-complex with various MoO_3 fractions. The Ni 2*p* peak is deconvoluted to Ni^{2+}, Ni^{3+}, Ni $2p_{3/2}$, shake up, and Ni $2p_{3/2}$ satellite peaks. **a** Pristine NiO, **b** NiO:MoO_3 15 at.%, **c** NiO:MoO_3 30 at.%, **d** NiO:MoO_3 50 at.%, **e** NiO:MoO_3 70 at.%, and **f** NiO:MoO_3 85 at.%.

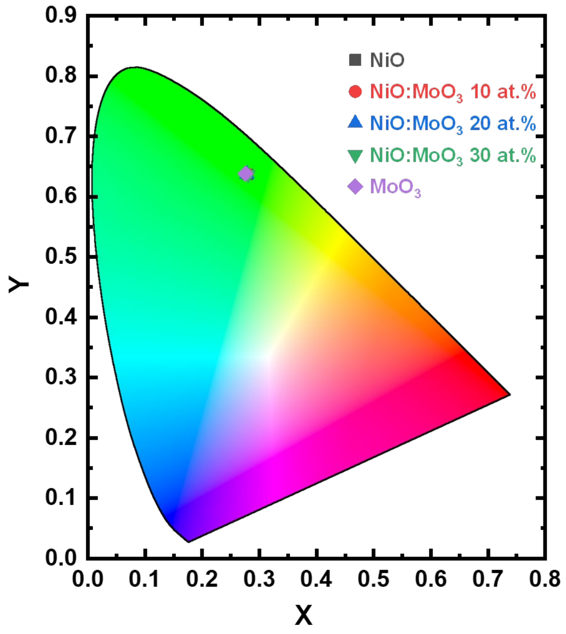

Supplementary Fig. 11. CIE 1931 coordinates for NiO, NiO:MoO_3-complex, and MoO_3 based green phosphorescent OLEDs.

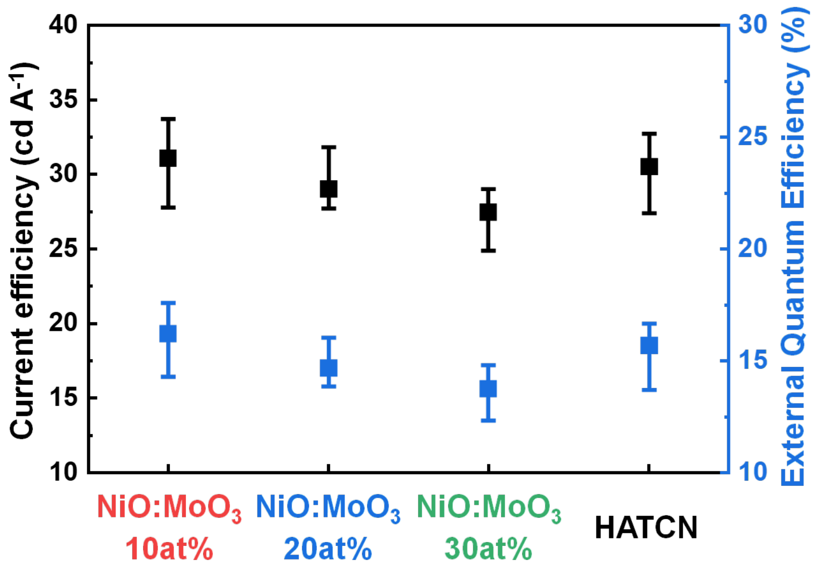

Supplementary Fig. 12. Comparison of peak current efficiency and EQE of blue phosphorescent OLEDs corresponding to 10, 20, 30 at.% of NiO:MoO₃, and HATCN HIL. Mean value of current efficiency was 31.1, 29.0, 27.5, and 30.5 Cd A^{-1} for NiO:MoO_3 10 at.%, 20 at.%, 30 at.%, and HATCN, respectively. Average value of EQE was 16.1, 14.6, 13.7, and 15.6% for NiO:MoO_3 10 at.%, 20 at.%, 30 at.%, and HATCN, respectively. Error bars were acquired from five OLED devices for each HIL type.

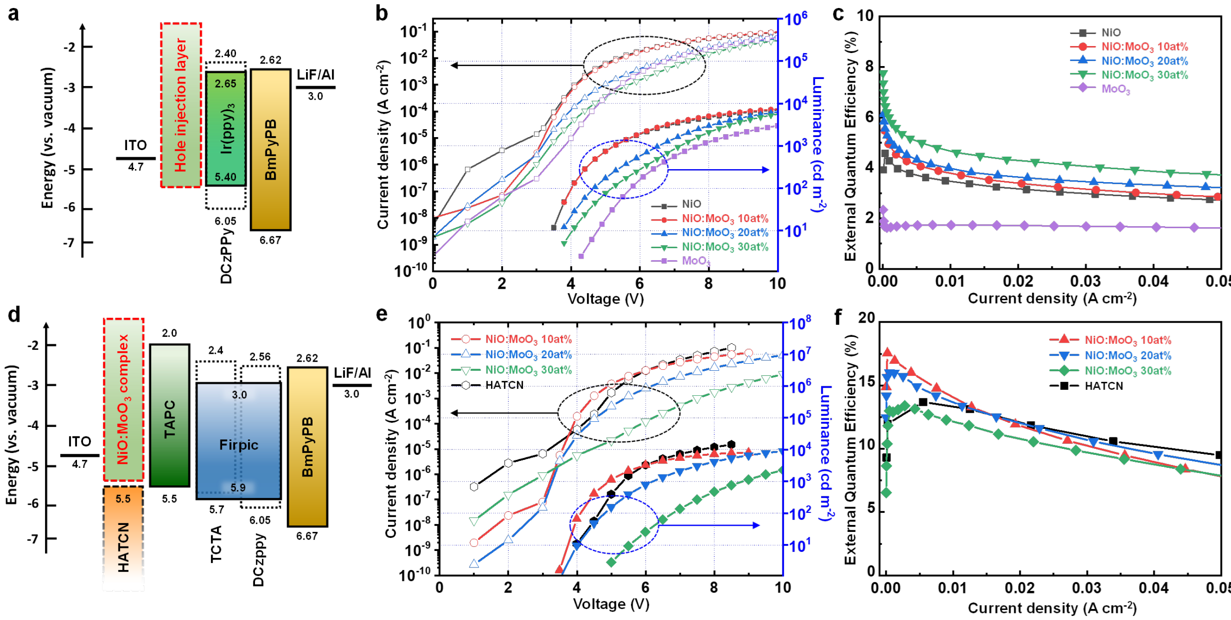

Supplementary Fig. 13. Detailed device characteristics of green and green and blue phosphorescent OLEDs. a Band structure of green phosphorescent OLED device. **b** Their logarithmic plot of *J-V-L* and **c** current density-EQE curves (relative to Fig. 4). **d** Band structure of blue phosphorescent OLED device. **e** Their logarithmic plot of *J-V-L* and **f** current density-EQE curves (relative to Fig. 5).

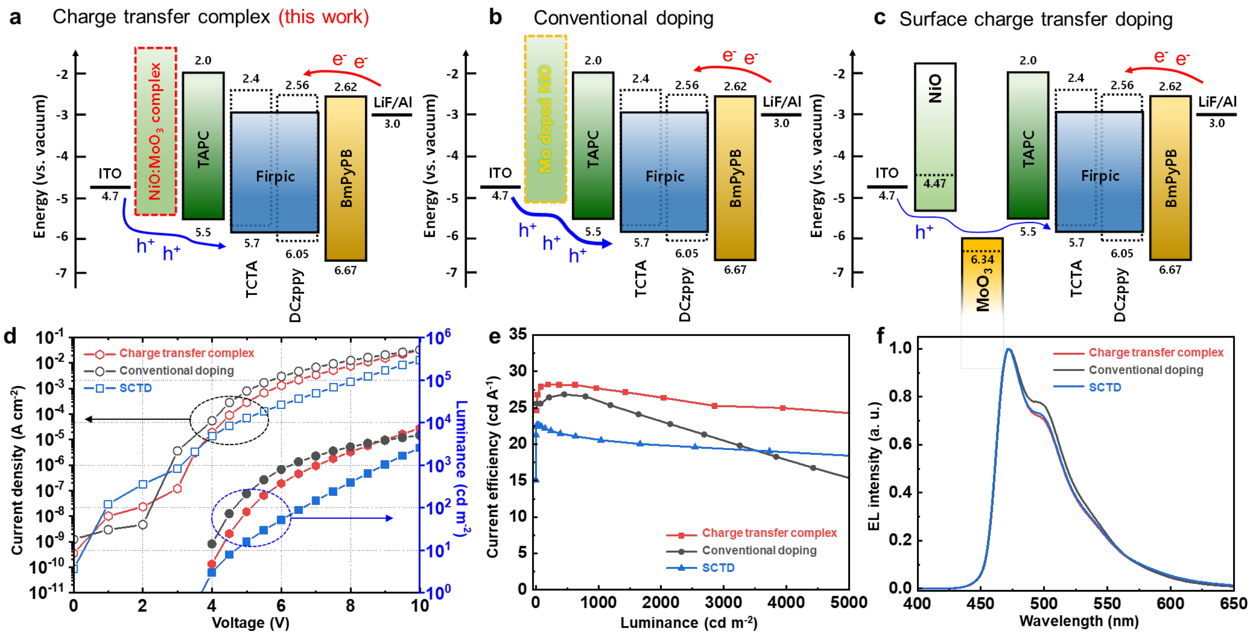

Supplementary Fig. 14. Comparison of metal oxide enhancing strategies in blue phosphorescent OLED system. Schematics of energy band and charge balance of **a** charge transfer complex (this work), **b** conventional doping, and **c** surface charge transfer doping (SCTD). **d** Current density-voltage-luminance curves, **e** luminance-current efficiency curves, and **f** normalized electroluminescence for devices prepared by charge transfer complex (NiO:MoO_3 10 at.%), conventional doping (NiO:Mo 10 at.%), and SCTD (NiO/MoO_3 bilayer).

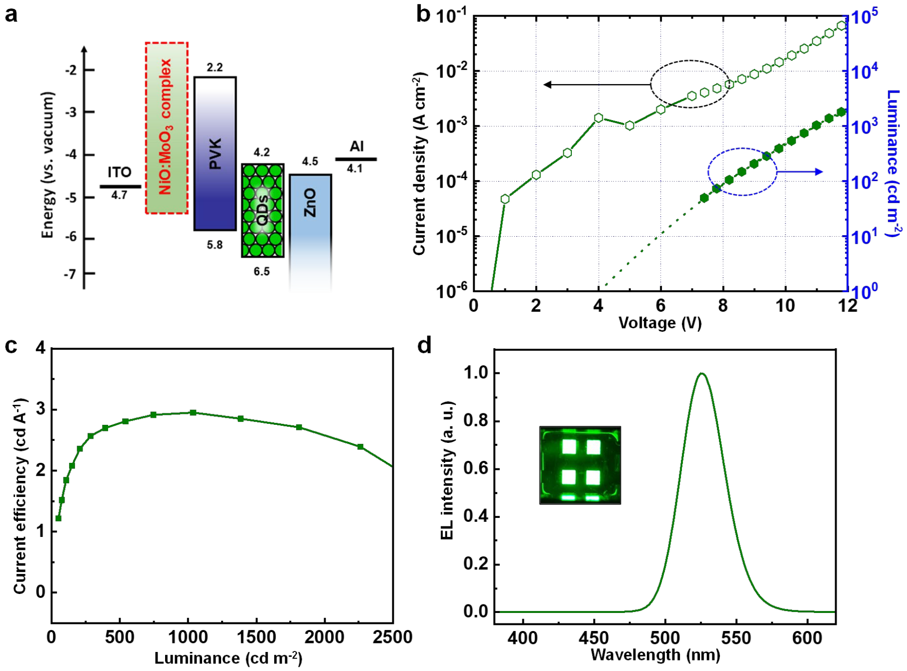

Supplementary Fig. 15. Characterization of quantum dot light emitting diodes (QLEDs). a Energy level diagram of multilayer QLED device. **b** Current density-voltage and luminance curves as a function of applied voltage, **c** luminance-current efficiency curves, **d** normalized electroluminescence for NiO:MoO_3-complex QLEDs. The dashed line in **b** is the polynomial fit curve of luminance. The inset image in **d** shows an operating QLED device.

Methods

QLED device fabrication.

The ITO-patterned glass substrates (resistivity: < 20 ohm square^{-1}) were cleaned by sonication with acetone, ethanol, and isopropyl alcohol for 30 min each, followed by UV-O_3 treatment for 20 min. The QLED devices were fabricated by spin-coating NiO:MoO_3 complex, PVK (TCI), QDs (Uniam), and ZnO in order, except for the Al cathode. After spin-casting of NiO:MoO_3 10 at.% complex (3000 rpm, 30 s), the sample was annealed at 300 °C for 1 hour under ambient conditions and was then moved to a N_2 gas filled glove box to form the next layers. PVK (8 mg mL^{-1} in chlorobenzene) was spin-coated at 3000 rpm for 60 s and baked at 180 °C for 30 min, followed by QD solution (15 mg mL^{-1} in octane) coating at 3000 rpm for 30 seconds and baking for 30 min at 80 °C. ZnO NPs (30 mg mL^{-1} in 2-methoxyethanol) were deposited by spin-coating (3000 rpm for 60 seconds) onto the QD layer. Finally, a 120 nm Al cathode was thermally evaporated under high vacuum conditions (10^{-8} mbar).

Synthesis of ZnO NPs.

To synthesize ZnO NPs, 1.8 mmol of zinc acetate dihydrate ($Zn(OAc)_2 \cdot 2H_2O$) was loaded in a 100 mL flask with 20 ml of ethanol. The Zn precursor solution was stirred for 1 h at 80 °C. After adding 2.8 mmol of lithium hydroxide monohydrate ($LiOH \cdot H_2O$), the flask was sonicated for 1 h to synthesize ZnO NPs. Finally, the synthesized ZnO NP solution was purified by centrifugation with hexane mixed into the solution.

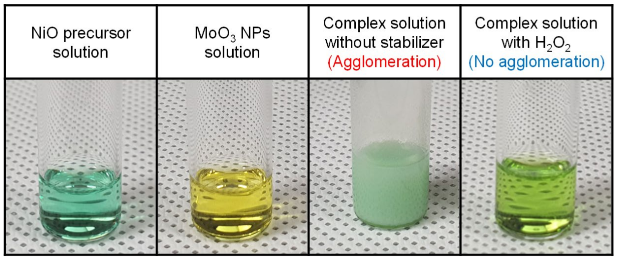

Supplementary Fig. 16. H_2O_2 as a stabilizer in NiO precursor and MoO_3 NP complexing process. The complex solution with 0.4 M of H_2O_2 as a stabilizer shows a clear green color while the simple blended solution was opaque and agglomerated. H_2O_2 is introduced to hinder agglomeration by providing extra -OH and -OOH ligands of MoO_3 NPs.

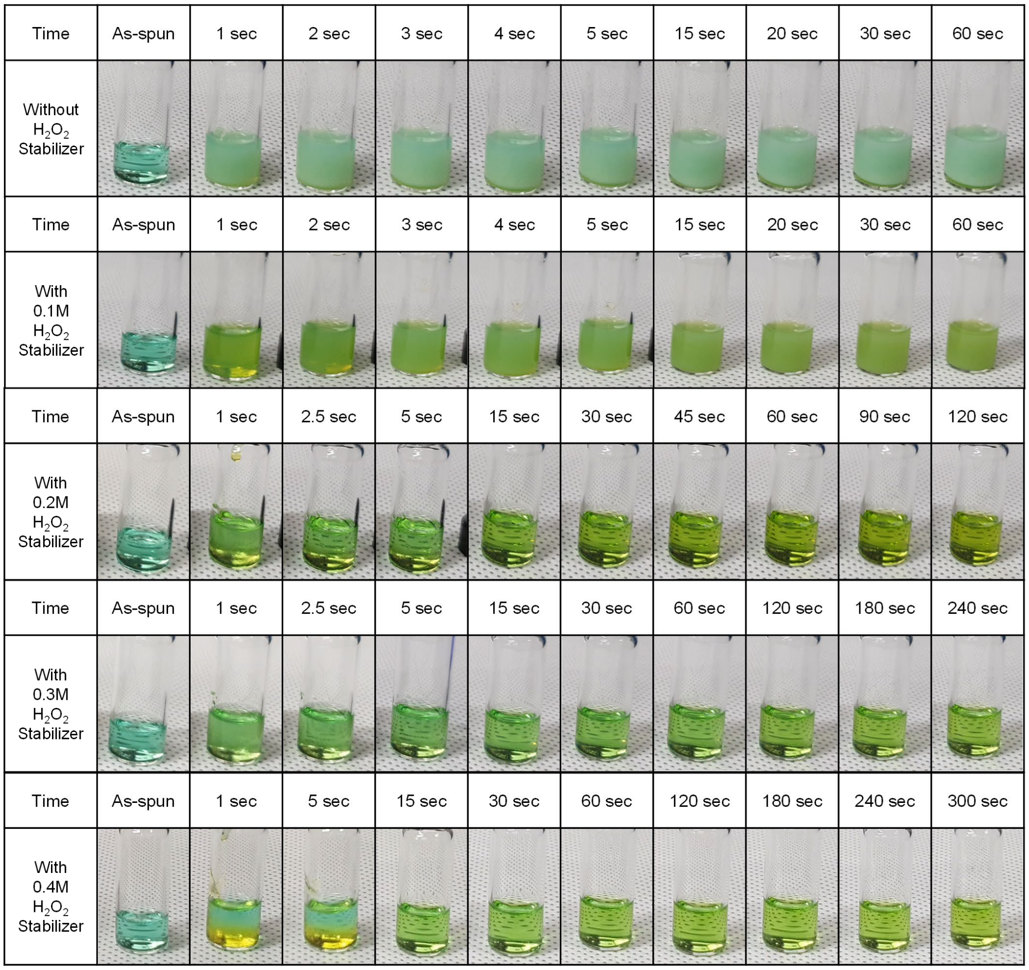

Supplementary Fig. 17. Stability of the complex solution depending on the added H_2O_2 concentration. Snapshots of Supplementary Movie 1 at certain times immediately after blending the two solutions are provided.

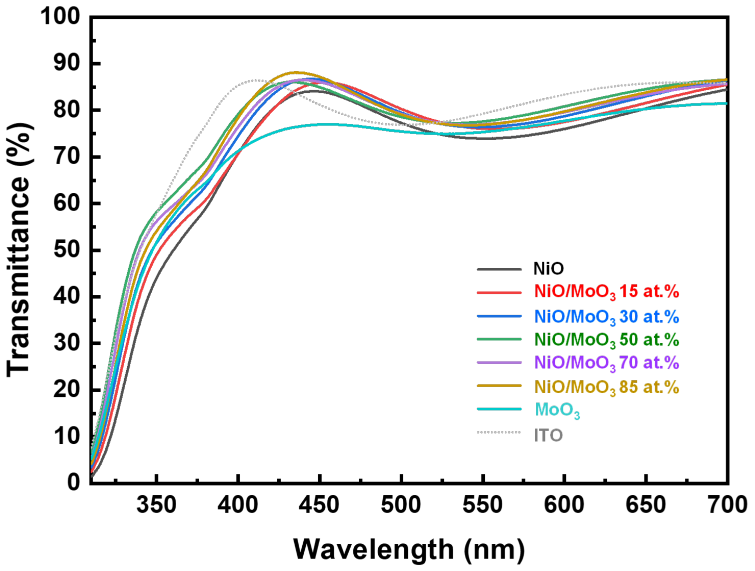

Supplementary Fig. 18. Transmittance spectra of NiO:MoO_3-complex with various MoO_3 fractions in NiO:MoO_3-complex and ITO substrate. NiO:MoO_3-complex films show increased transmittance in the visible range (400-700 nm) compared to pristine NiO and MoO_3 due to NiO amorphization.
