# Supplementary material for: Metal oxide charge transfer complex for effective energy band tailoring in multilayer optoelectronics
Source: Nat Commun. 2022 Jan 10;13:75. doi: 10.1038/s41467-021-27652-3 (PMC8748812; doi:10.1038/s41467-021-27652-3)
Supplement: Supplementary file 3 — Description of Additional Supplementary Files [file 41467_2021_27652_MOESM3_ESM.pdf]

File Name: Supplementary Movie 1

Description: Control of agglomeration in the NiO:MoO3- complex solution by addition of H2O2 stabilizer.
